# Participant Compliance With Ecological Momentary Assessment in Movement Behavior Research Among Adolescents and Emerging Adults: Systematic Review

**DOI:** 10.2196/52887

**Published:** 2025-02-11

**Authors:** Shirlene Wang, Chih-Hsiang Yang, Denver Brown, Alan Cheng, Matthew Y W Kwan

**Affiliations:** 1 Department of Population and Public Health Sciences, University of Southern California Los Angeles, CA United States; 2 Department of Preventive Medicine, Northwestern University Chicago, IL United States; 3 Department of Exercise Science and TecHealth Center, University of South Carolina Columbia, SC United States; 4 Department of Psychology, The University of Texas at San Antonio San Antonio, TX United States; 5 Department of Child and Youth Studies, Brock University St. Catherines, ON Canada; 6 Department of Family Medicine, McMaster University Hamilton, ON Canada

**Keywords:** compliance, ecological momentary assessment, mobile health, adolescents, emerging adults, physical activity, movement behavior, systematic review, cognitive, social, development, youth, literature search, inclusion, data quality, mobile phone

## Abstract

**Background:**

Adolescence through emerging adulthood represents a critical period associated with changes in lifestyle behaviors. Understanding the dynamic relationships between cognitive, social, and environmental contexts is informative for the development of interventions aiming to help youth sustain physical activity and limit sedentary time during this life stage. Ecological momentary assessment (EMA) is an innovative method involving real-time assessment of individuals’ experiences and behaviors in their naturalistic or everyday environments; however, EMA compliance can be problematic due to high participant burdens.

**Objective:**

This systematic review synthesized existing evidence pertaining to compliance in EMA studies that investigated wake-time movement behaviors among adolescent and emerging adult populations. Differences in EMA delivery scheme or protocol, EMA platforms, prompting schedules, and compensation methods—all of which can affect participant compliance and overall study quality—were examined.

**Methods:**

An electronic literature search was conducted in PubMed, PsycINFO, and Web of Science databases to select relevant papers that assessed movement behaviors among the population using EMA and reported compliance information for inclusion (n=52) in October 2022. Study quality was assessed using a modified version of the Checklist for Reporting of EMA Studies (CREMAS).

**Results:**

Synthesizing the existing evidence revealed several factors that influence compliance. The platform used for EMA studies could affect compliance and data quality in that studies using smartphones or apps might lessen additional burdens associated with delivering EMAs, yet most studies used web-based formats (n=18, 35%). Study length was not found to affect EMA compliance rates, but the timing and frequency of prompts may be critical factors associated with missingness. For example, studies that only prompted participants once per day had higher compliance (91% vs 77%), but more frequent prompts provided more comprehensive data for researchers at the expense of increased participant burden. Similarly, studies with frequent prompting within the day may provide more representative data but may also be perceived as more burdensome and result in lower compliance. Compensation type did not significantly affect compliance, but additional motivational strategies could be applied to encourage participant response.

**Conclusions:**

Ultimately, researchers should consider the best strategies to limit burdens, balanced against requirements to answer the research question or phenomena being studied. Findings also highlight the need for greater consistency in reporting and more specificity when explaining procedures to understand how EMA compliance could be optimized in studies examining physical activity and sedentary time among youth.

**Trial Registration:**

PROSPERO CRD42021282093; https://www.crd.york.ac.uk/prospero/display_record.php?RecordID=282093

## Introduction

The transition from adolescence (aged 10-18 years) to emerging adulthood (aged 18-29 years) represents a critical developmental period [1] that is often associated with significant changes in lifestyle behaviors [2-4]. To establish more effective interventions or programs to help youth (ie, adolescents and emerging adults) promote or sustain more physical activity (PA) and limit sedentary time (ST), there has been growing interest in studying the dynamic relationships between youths’ cognitions, social and environmental contexts, and movement behaviors. Research has consistently shown greater PA and less ST being beneficial for several health outcomes for both adolescents and emerging adults including self-reported health status, chronic diseases such as obesity and cardiovascular disease, and indicators of mental health [5,6]. Ecological momentary assessment (EMA) is an innovative method for collecting intensive longitudinal data to better understand the dynamic predictors of these critical health-promoting behaviors [7]. In brief, EMA involves repeated real-time assessment of individuals’ experiences and behaviors in their real world and natural environments, which do not require participants to recall extended periods of time, helping to reduce participant recall biases and increase ecological validity [8]. These methods have been implemented in several studies to date, helping to advance knowledge related to real-time correlates of PA and ST among youth. While there has been considerable progress in this field, EMA compliance can, at times, be problematic among the youth population. Our understanding of EMA compliance among movement behavior studies remains poor and thus warrants greater attention to help inform the development of future and more effective EMA protocols.

Data collection efforts involving repeated participant observations such as EMA allow researchers to study the temporal patterns and momentary processes that influence movement behaviors while allowing for the disaggregation of data into between-subjects and within-subjects for analysis [9]. EMA methods have evolved over time, beginning with more traditional methods such as paper and pencil diaries and repeated telephone calls, to the integration with technology using palm computers or SMS text messaging, and now smartphone apps developed for EMA [10]. There are several sampling approaches used in EMA, each suited to different types of research questions and contexts. In signal-contingent sampling, participants are prompted to provide data at random or within predetermined intervals. In contrast, data are only collected after the occurrence of specific events in event-contingent sampling. Given the ubiquity and rapid technological advances of smartphones to reduce some of the participant burden, it is not surprising that EMA research among adolescent and emerging adulthood populations has risen over recent years. Delivering EMA protocols through smartphones is often considered a feasible and acceptable method to collect intensive longitudinal data among youth samples due to their high rates of device use [11,12]. In other words, the utilization of smartphones has the unique potential to improve our understanding of correlates of PA and ST, but poor adherence to the EMA protocol may bias the associations observed, ultimately hindering opportunities to develop effective interventions.

Despite the aid of technology, EMA using smartphones remains a burden-heavy method for data collection for participants. Its repeated self-report nature requiring conscious effort lends itself to potentially substantive missing data as answering prompts in naturalistic settings may not always be feasible. For example, participants may not be able to answer an EMA prompt in a timely manner due to engagement in sport or PA, being prompted in unsafe contexts (eg, while driving), or if the prompt was sent at an inconvenient time (eg, socializing with friends or napping). For these reasons, it is understood that missingness in EMA research is inevitable as an increased burden leads to lower study compliance. However, missing data that results from a low EMA response rate raises questions about the quality of the data being collected. Despite its time-consuming nature, compliance with EMA is essential for the validity or representativeness of daily phenomena that may influence movement behaviors among youth [13].

Compliance is commonly defined as the ratio of the number of measurement occasions participants completed divided by the maximum number of measurement occasions allowed by the protocol of the EMA study [14]. Conducting an EMA study involves multiple decision-making processes to determine the protocol, including but not limited to the EMA assessment tool and platform, compensation, study duration, assessment frequency, sampling scheme, and questionnaire density [15]. Missingness and low compliance can impact the data quality and be susceptible to misinterpretation. For example, systematic missingness could introduce bias as the resulting data does not reflect the true distribution of behaviors. Similarly, the overall sample may not represent the intended population and limit generalizability. Missing data also reduces sample size, which decreases the statistical power of the study or increases the variability of estimates. Finally, if there is missing data at critical times (eg, ignoring prompts during stressful times or missing prompts because they are engaging in PA within a PA study), the trends could be misinterpreted. While there are techniques to impute missing data, imputing EMA data can be challenging due to the inherent dynamic nature of the data and lead to inaccurate results. Given that compliance is critical to understanding the representativeness of the collected data and earlier publications pushing for compliance reporting for EMA studies [7,16,17] it is crucial to understand the factors and study methodological features that may be influencing EMA compliance.

Indeed, studies have examined the application of EMA in youth. Previous reviews have focused on summarizing or reporting the main characteristics of studies that apply EMA to study mental health or psychopathology in daily life. A paper by Mason et al [18] examined the association between EMA-measured contextual factors and dietary intake in youth. Liao et al [16] conducted a systematic review of common EMA methods of diet and PA research in youth. In a previous systematic review of EMA protocol and compliance in adolescents, Wen et al [12] concluded that compliance was similar for studies longer than 3 weeks compared to studies less than 1 week. Interestingly, another systematic review of EMA studies in the adult population found no identified associations between any features of the EMA protocol and the compliance rate [17]. Given that youth populations may not fully understand what would be required in an EMA study, or can also be generally ambivalent toward research, it is crucial to understand potential methods and practices that may result in greater compliance. The existing reviews included EMA studies that focused on a wide range of health outcomes, which makes it difficult to disentangle the potential impact of EMA protocols for specific behavioral outcomes. Movement behaviors, which can include PA and sedentary behaviors may be particularly unique, as the continual collection of movement behavior data through self-reports or device-based assessments may represent an additional source of participant burden. A more nuanced understanding of EMA compliance in movement behavior studies is needed. In addition, compliance cannot be solely interpreted without considering the quality of EMA design, as poor design features also lead to a greater burden and decreased compliance. Suggested guidelines for reporting EMA study protocol, such as the Checklist for Reporting of EMA Studies (CREMAS) framework, have been adopted by EMA researchers to evaluate the quality and report the design features in a standard manner [16].

Therefore, the purpose of this systematic review was to apply the CREMAS guidelines to (1) synthesize the existing evidence on compliance for EMA studies focusing on wake-time movement behaviors (ie, PA and ST) among adolescent and emerging adult populations, and (2) evaluate the quality of the included EMA studies. We summarize the repeated measure methodologies used when examining PA and ST behaviors and describe any salient factors that may positively or negatively impact EMA compliance.

## Methods

### Protocol and Registration

Before conducting this systematic review, a protocol was developed and preregistered on PROSPERO (submitted October 21, 2021; registration number CRD42021282093). The PRISMA (Preferred Reporting Items for Systematic Reviews and Meta-Analyses) guidelines were followed [19], and items were reported using the PRISMA checklist (Multimedia Appendix 1).

### Search Strategy

An electronic literature search was conducted in the PubMed, PsycINFO, and Web of Science databases on October 19, 2022. First, titles and abstracts of papers were searched using the following terms: (“ambulatory assessment” OR “experience sampling” OR “ecological momentary assessment” OR “EMA” OR “ESM” OR “daily diary”) AND (“physical activity” OR “exercise” OR “sedentary” OR “movement behavior” OR “movement-based behavior” OR “posture” OR “sport” OR “sitting” OR “screen” OR “inactivity” OR “leisure activity”) AND (“young adult*” OR “youth” OR “college student*” OR “university student*” OR “emerging adult*” OR “adolescence” OR “adolescent” OR “high school students” or “secondary school students” OR “post secondary students”). The same search strategy was used across all 3 databases.

### Inclusion and Exclusion Criteria

We included studies that met the following criteria: (1) published in a peer-reviewed academic journal, (2) full text was available, (3) published in the English language, (4) delivered EMA prompts at least one time each day during the study period (minimum requirement for daily diary studies), (5) provide quantitative data for movement-based activity (ie, PA or ST; to exclude protocol papers), (6) provide quantitative compliance data (% or number of completed) for EMA prompt responses, and (7) the sample consisted of adolescents or young adults (aged 10-29 years). Papers were excluded if (1) the population included a mix of participants older or younger than the target age range (ie, <10 or >29 years of age), (2) the study design was an intervention, (3) the paper reported on multiple studies that used EMA, or (4) the paper was not the first study to publish using a particular data set (ie, an EMA study was only counted once when multiple publications came from the same EMA project; the first publication was included in these instances so each study protocol was only represented once).

### Study Selection

All records found from the database searches were imported into Covidence systematic review software (Veritas Health Innovation). Duplicates were removed before titles and abstracts were screened independently by 2 reviewers (SW and AC) for initial inclusion. All studies retained after the title and abstract screening were retrieved as full papers and further assessed for final inclusion by 2 reviewers independently. Disagreements at both screening stages were resolved by discussion during review team meetings.

### Data Extraction and Synthesis

For all studies that met our inclusion criteria, the lead author (SW) extracted the following data into an Excel (Microsoft Corp) data entry form: (1) authors and year of publication; (2) sample characteristics (ie, sample size, country, mean age of sample); (3) EMA device or platform; (4) types of movement activity that was assessed, including whether it was self-reported or device-measured; (5) coassessment of diet; (6) study duration; (7) prompting schedule (event or time or signal contingent); (8) prompt frequency; (9) number of items for each prompt; (10) prompt times; (11) compensation or types of incentive; and (12) reported details about compliance (rate, inclusion criteria, and missing data relationships). Multimedia Appendix 2 [20-68] presents the extracted data. In addition, a random sample of 20% of the included papers was independently extracted by a second review team member (AC) for validation (interrater reliability >80%). The compliance rate was calculated for studies that reported the total number of surveys completed or the average number of surveys completed by dividing by the total number of expected delivered surveys as reported by the prompting scheme in the paper’s methods. Given the population, many studies had different prompting scheduled for school days versus weekend days. Therefore, we considered the maximum or most burdensome day when calculating the density of prompts (number of prompts per day).

A quality score for each study was calculated using a modified version of the Checklist for Reporting of EMA Studies (CREMAS) [16]. The checklist was used to assess if descriptions were provided for EMA technology, EMA protocol training, study duration, prompting scheme, prompting frequency, study attrition, prompt latency, missing data, and limitations of EMA. Each item was scored with 0, 0.5, or 1 (representing No, Somewhat, Yes). Scores ranged from 0 to 9, with greater scores representing higher quality. Scores can be found in Multimedia Appendix 2 [20-68].

Given the heterogeneity in the samples and methods in the studies that met the inclusion criteria, further analysis through conducting a meta-analysis was not appropriate. We proceeded with our objective of conducting a narrative synthesis, and studies were reviewed and compared on a variety of characteristics, including PA/ST assessment method, EMA platform, number of study days, prompting schedule, prompting frequency, number of prompts per day, compensation, and the comeasurement of diet. Differences in compliance between groups in each category were analyzed using Kruskal-Wallis tests. Additionally, potential interactions between significant factors were presented in contingency tables as many of the factors we examined could coexist with each other to compound burden (EMA delivery scheme × study duration × prompt frequency). For example, very intensive prompting (multiple times per day) may only be tolerable for a short period of time in comparison to end-of-day prompts completed 1 time per day.

## Results

### Overview

**Figure 1:**
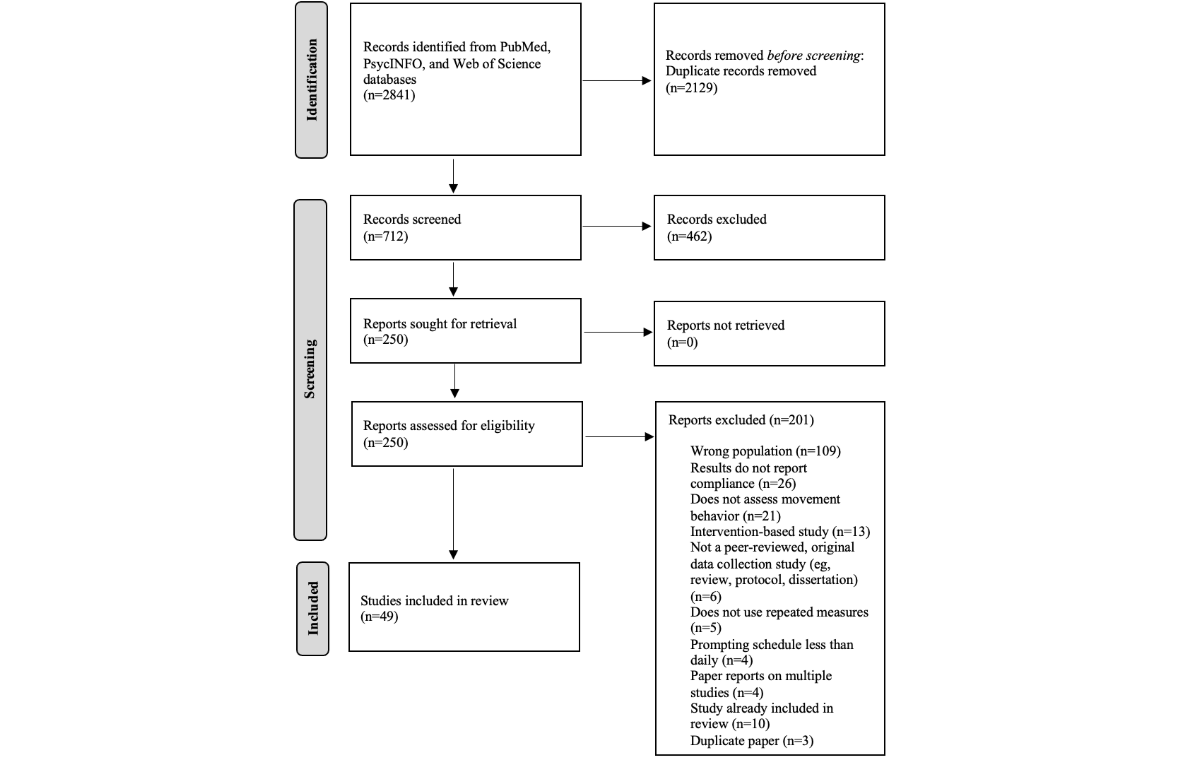
Adapted PRISMA flow diagram for identification and selection of studies. PRISMA: Preferred Reporting Items for Systematic Reviews and Meta-Analyses.

Our systematic search conducted at 2 time points (ie, November 2021 and October 2022) captured a total of 2841 papers, of which 2129 papers were duplicates and therefore removed. A total of 712 remaining papers were initially screened. After title and abstract screening, an additional 462 papers were deemed irrelevant, with the remaining 250 papers proceeding for full-text screening. Following the full-text review, 201 papers were found to have not met the eligibility criteria, resulting in 49 papers retained for data extraction and included in this review [20-68]. A PRISMA diagram for selecting the final papers for synthesizing is presented in Figure 1.

### Study Quality

Table 1 presents the quality scores of the final papers. The average CREMAS score was 6.84 (SD 1.26). All studies reported (yes or somewhat) the study duration, prompting scheme, and prompting frequency. However, studies most infrequently reported on if and how participants were trained on the EMA protocol (n=18, 37% not reported), how missing data were handled (n=28, 57% not reported), attrition (n=26, 53% not reported), and latency (n=42, 86% not reported). These results highlight the continued variability in reporting critical attributes within PA and ST research among adolescents and emerging adults when applying EMA methodologies.

**Table 1:** Study (n=49) quality scores based on the adapted CREMAS^a^ tool.

CREMAS item	Yes, n (%)	Somewhat, n (%)	No, n (%)
EMA^b^ technology	35 (71)	12 (25)	2 (4)
EMA^b^ training	23 (47)	8 (16)	18 (37)
Study duration	48 (98)	1 (2)	0 (0)
Prompting scheme	47 (96)	2 (4)	0 (0)
Frequency	48 (98)	1 (2)	0 (0)
Attrition	11 (22)	12 (25)	26 (53)
Latency	3 (6)	4 (8)	42 (86)
Missing data	19 (39)	2 (4)	28 (57)
Limitations of EMA^b^	26 (53)	10 (20)	13 (27)

^a^CREMAS: Checklist for Reporting of EMA Studies.

^b^EMA: ecological momentary assessment.

### Descriptive Statistics

PA was the primary outcome in 40 (82%) of the 49 studies. More studies used self-reports of PA and ST (n=29, 59%) than device-based assessments (n=20, 41%) to measure movement behaviors. For compensation, 9 (18%) studies provided some form of course credit, 12 (24%) studies provided only monetary compensation, and 5 (10%) studies provided both options. In contrast, 1 study explicitly stated that no compensation was provided for participation, and another 22 (45%) of the 49 studies did not report how participants were compensated.

Although most studies were conducted in the United States (n=36, 73%) among nonclinical samples (n=46, 94%), there was substantial heterogeneity in the EMA study methodologies. For example, the sample size of these studies ranged from 8 to 1493 participants (mean 179.77, SD 272.43), study durations from 1 to 30 days (including 4 studies that used multiple waves of EMA data collection periods [29,48,53,61]), and a variety of prompting schedules being applied. A total of 12 (24%) studies consisted of daily dairies, whereby participants were asked to respond to a single EMA prompt once each day [27-29,31,35,39-41,54,59,62,66]. Another 36 (73%) studies involved multiple prompts each day, including 4 studies that used event-contingent prompting [20,36,44,65]. These 4 studies required participants to self-report eating episodes [65], or the prompts were triggered by their current location [44] or current activity [20,36]. Among the 32 studies with only a time-based sampling component (predetermined sets of prompts being administered), prompting schedules included random prompts during predetermined time intervals (n=30, 61%; eg, one random prompt for each 2-hour interval), prompts at a fixed schedule (n=8, 16%; eg, every 30 min during waking hours), or prompts at a personalized time (n=1, 2%; eg, participant’s blood glucose check schedule). One study did not report the prompting scheme. A total of 13 (27%) of the 49 studies did not report the length of the EMA surveys. Of the studies that were reported, the number of items per prompt ranged from 1 to 45 (mean 11.78, SD 8.80). There was a negative but nonsignificant correlation between the length of the survey and compliance rate (*r*=–0.10; *P=*.57).

### Compliance Results

Overall, compliance data were extracted for 48 studies, as we could not extract the compliance rate for 1 study that reported the average number of event-contingent prompts responded to [64]. The compliance rate was calculated for studies that reported the total number of surveys completed or the average number of surveys completed by dividing by the total number of expected delivered surveys as reported by the prompting scheme in the paper’s methods. Importantly, it should be noted that 13 (27%) of the 48 studies reported dropping participants from their analyses—which invariably improved calculations of compliance. Decision rules to exclude participants in final analyses varied, with various thresholds of minimum compliance ranging from 30% to 80% missing data or removal of participant data if compliance rates were beyond 2-3 standard deviations. It may also be possible that the remaining 35 studies also removed participant data without being specified. Among the 48 studies with reported compliance, the mean compliance was 80.63% (range 40.2%-99.3%). The mean compliance, however, was higher among the daily diary studies (91.03%; range 88.3%-96%) than studies with multiple prompts each day (77.16%; range 40%-95%).

Overall, mean compliance among the daily diary studies was relatively high and consistent across studies. Compliance was greater than 88% in all studies, with study durations ranging from 6 to 30 days of data collection. Among the 12 daily diary studies, 10 studies reported using web-based platforms, including 1 study that also used paper and pencil reporting. One study was phone-based, and 1 study did not report the modality of EMA collection. All studies were published after 2013, but 9 of these daily diary studies were published within 5 years of this conducted review.

The mean compliance among studies with multiple prompts per day was more variable when compared to daily diary studies, with reported compliance as low as 40% to as high as 94%. In examining prompt type and duration of EMA studies, there were no distinct patterns to compliance based on whether studies applied fixed or random prompting scheduled duration of the EMA studies. There is more variability when examining the interactions between prompt schedules and study duration (range 71.45%-90%), but with a caveat that some include only a small number of studies (eg, only 2 studies that used a fixed prompting schedule for less than 1 week).

With regard to prompt schedules, among these multiple EMA prompt studies, there was a substantial number of studies (n=8) that included very high numbers of EMA surveys being delivered per day (range 17-68). Interestingly, the mean compliance of these studies was higher (82.10%; range 50.20%-99.30%) when compared to studies (n=27) that included 3 to 9 prompts per day (76.43%; range 40%-89.30%). Two studies did not specify the number of prompts they sent each day. Studies with the most intensive EMA protocols (ie, ≥17 prompts per day) had compliance of greater than 82%, apart from 1 study with 50% compliance. The highest compliance rate (99%) was reported by Kanning et al [50], who intensively delivered EMA prompts every 45 minutes for 1 day to study the real-time association of PA with affect. Three studies used extra devices to send electronic EMA surveys, while 4 studies used paper and pencil EMA methods.

Upon closer examination of studies prompting participants between 3 and 9 times each day, 19 of the 27 studies were found to have had compliance above 75%. Interestingly, 2 studies used paper and pencil as the modality of EMA collection; however, both studies had compliance between 76% and 77%. Studies with lower compliance varied in sample size (ie, 20 to 284 participants), study duration (ie, 4 to 30 days), prompting schedules, forms of compensation, and year of publication (ie, 2013-2022).

### Factors Related to Compliance

Due to the small sample size of studies in various categories of synthesizing, nonparametric Kruskal-Wallis tests were performed to determine the effects of the factors on median compliance rates. These results are presented in Table 2. The results indicated nonsignificant differences for the movement behavior assessment method (*χ*^2^_2_=0.2; *P*=.88); number of items per prompt (*χ*^2^_3_=1.2; *P*=.75); number of days (*χ*^2^_3_=1.1; *P*=.77); or compensation structure (*χ*^2^_4_=3.9; *P*=.42). There was a statistically significant difference between compliance based on prompting frequency (*χ*^2^_3_=15.2; *P*<.001) and the binned number of prompts per day (*χ*^2^_3_=1.2; *P*<.001). Furthermore, a statistically significant difference was observed for compliance based on the prompting schedule (*χ*^2^_4_=9.9; *P*=.04) and compliance based on the EMA platform (*χ*^2^_5_= 9.5; *P*=.04). Additional linear regression analyses did not find a statistically significant relationship between the number of study days and the number of prompts per day (coded as continuous variables) with compliance. There was no statistically significant difference in mean compliance between the studies that only used objective assessments (n=15; 81.57%) versus self-reported PA (n=33; 80.20%; t_46_=–0.11; *P*=.46). There was no statistically significant difference in compliance between the studies that had PA as the primary outcome (40/48, 81.22%) versus not (8/48, 77.66%; t_46_=–0.23; *P*=.41).

**Table 2:** Comparisons of compliance by EMA^a^ study design factors.

Factor		Value, n	Median compliance	Rank sum	Chi-square (*df*)		*P* value	
**PA^b^/ST^c^ assessment**						0.2 (2)		.88
	Device assessed	15	81.57	381.5				
	Self-report	29	80.25	688.5				
	Both	4	84.70	106				
**EMA platform**						11.4 (5)		.04
	App on personal device	8	75.21	132.5				
	Extra device	13	79.29	278				
	Paper and pencil	7	74.60	127.5				
	Telephone call	2	80.00	53				
	Web-based	16	86.15	503				
	NR^d^	2	91.33	82				
**Days**						1.1 (3)		.77
	<7	9	78.37	226.5				
	7-14	21	81.27	527				
	14-28	13	83.10	331				
	>28	5	75.56	91.5				
**Prompting schedule**						9.5 (4)		.04
	Event	2	79.25	30				
	Event + interval	2	81.60	40				
	Random	9	80.10	178				
	Interval	27	83.95	805				
	Random + interval	8	70.14	122				
**Prompting frequency**						15.2 (3)		<.001
	Daily	12	91.03	456.5				
	Event + multiple	3	80.17	51				
	Multiple	32	76.72	646				
	NR^d^	1	82.5	22.5				
**Number of prompts per day**						17.7 (3)		<.001
	1	12	91.03	394.5				
	2-4	7	77.23	119				
	4-10	19	77	306.5				
	>10	4	80.65	83				
**Number of items per prompt**						1.2 (3)		.75
	0-10	16	79.54	301				
	11-20	15	78.33	293				
	21-30	4	82.95	86				
	41-50	2	73.51	23				
**Compensation**						3.9 (4)		.42
	Course credit	9	81.34	235.5				
	Monetary	12	80.34	250.5				
	Both	5	80.90	112.5				
	None	1	99.30	48				
	NR^d^	21	79.53	529.5				

^a^EMA: ecological momentary assessment.

^b^PA: physical activity.

^c^ST: sedentary time.

^d^NR: not reported.

## Discussion

### Principal Findings

This systematic review addressed a current knowledge gap by synthesizing the body of literature that has used EMA methodologies to investigate adolescents’ and emerging adults’ movement behaviors for the purpose of identifying protocol-related factors that influence EMA compliance. Only 1 previous study has specifically examined factors related to compliance among youth. Heron et al [11] conducted a systematic review to provide best practices by identifying common approaches and challenges and providing reporting guidelines for compliance; however, do not specifically identify factors besides recommendations for staff to complete compliance check-in and implement compliance-based incentives. While there is substantial heterogeneity among the included EMA studies, the pooled compliance rate was high and comparable to that reported in previous reviews. Unlike the few findings in previous literature, this review identified several specific EMA design factors based on the CREMAS checklist (ie, delivery scheme, study duration, and prompt frequency), including factors that interact to influence participants’ compliance in responding to EMA surveys. It should be noted, however, that the disparities in compliance attributable to EMA design factors were rather small as this population surprisingly responded consistently to prompts. This being said, there were no major discrepancies based on the factors we analyzed that led to considerable missingness.

### Associations Between PA Assessment Tool and Compliance

A total of 20 studies measured PA using an accelerometer which may have reduced self-report biases and recall errors attributable to self-reported instruments [69] but potentially increased the burden associated with the study overall. We observed no significant difference in EMA compliance based on the PA assessment method. This suggests that compliance rates to EMA are not better when participants must wear a separate device (but do not have to self-report their PA levels through EMA). Despite the potential additional burden of wearing an accelerometer (uncomfortable, unfashionable, or inconvenient), participants did not reduce response rates to EMA.

### Associations Between EMA Duration, Prompting Schedule, and Compliance

Overall, there was no difference in compliance between schedules of different study lengths. Most studies lasted between 7 and 14 days (n=21). Our findings suggest that the timing and frequency of EMA prompts may be a more critical factor in EMA design when attempting to optimize compliance. Logically, participants are more likely to respond if prompts align with their schedule or if they occur at predictable intervals. Therefore, the frequency of EMA prompts must be balanced to minimize the burden while providing researchers with sufficient representative EMA data for analysis. At the same time, longer study durations (eg, ≥14 days) may provide more representative data on participants’ behavior and experiences but can be perceived as more burdensome and result in noncompliance. One potential explanation could be that participants have incorporated EMA into their routines, and the behavior of responding has become a habit. It is important to acknowledge, however, that while participants may initially be able to handle the prompting schedule, longer EMA studies may be subject to temporal influences like schedule changes (eg, summer break for adolescents and transition to another work schedule for emerging adults) that could threaten compliance rate.

Ultimately, researchers should consider the minimum required number of days or prompts per day necessary to answer their research question based on the nature of the phenomena being studied. How frequently are movement behaviors or the predictors of the behavior expected to change? These considerations need to be weighed against participants’ daily schedules and routines. For example, some studies involving adolescents intentionally did not prompt participants during school hours, which stands to be missed when this population tends to have opportunities to be physically active (eg, physical education, clubs, and athletics) [70]. Researchers could take this further and use adaptive sampling techniques to adjust the frequency based on contextual factors such as weekday school attendance or prompting participants between structured class times (ie, recess time or transitions between classes). In addition, most studies used only an interval-based design (n=27). These studies had the highest compliance, which supports the assumption that receiving prompts at consistent intervals is less disruptive than prompts at random and unpredictable times. Additional consideration may include the use of passive-sensing (eg, wearable monitoring) in conjunction with EMA or active-sensing, requiring participants to respond to prompts being delivered.

### Associations Between the EMA Platform and Compliance

We found that the platform used to deliver the EMA study or protocol could significantly impact compliance and data quality. In our sample of studies, most studies (n=16) used a web-based format (Qualtrics survey) to administer EMA. However, we did not disentangle how the link was provided to participants. For example, some researchers may have emailed the link, whereas others sent an SMS text message to participants. Both options still capitalize on the convenience of mobile devices. While carrying an extra device (n=13) may have been initially novel to youth, it may be considered burdensome now, especially in studies that require participants to wear an accelerometer to capture movement behavior. Due to the increased prevalence of smartphone ownership in adolescents, it is expected that EMA researchers will adopt smartphones or smartphone apps to lessen the additional burdens associated with delivering EMAs. Within smartphone apps, it is worth considering additional factors that may influence compliance, such as the user interface or design of the platform or the use of gamification aimed at increasing engagement [71]. Intensive self-monitoring is becoming more acceptable or feasible, especially in studying health and mental health, and there is evidence of youth and emerging adults using self-care health-tracking apps in daily life with high engagement [72]. But beyond wearable devices, the adoption of self-monitoring motivating factors is still in the early stages of PA research [70].

### Associations Between EMA Response Frequency and Prompts Per Day and Compliance

The adoption and maintenance of movement behaviors in youth are complicated as there is a combination of factors with different possible temporalities. Due to the feasibility and to support more advanced theories, more studies have included multiple prompts per day (ie, 12 daily diary studies vs 36 studies that prompted numerous times per day). However, we observed that the daily diary studies had higher compliance. While more frequent prompts provide more comprehensive data for researchers, this also increases participants’ burdens. Frequency should depend on the research question, the population being studied, and the EMA platform being used.

### Associations Between Participant Compensation Type and Compliance

Most included studies did not adequately report their compensation mode or protocol (n=21). Based on the studies that did report compensation type, there was no difference between providing monetary compensation or course credit. With monetary compensation, there are also different levels and the potential need to determine a rate per unit of burden, which is beyond our analyses. Beyond incentives, researchers should consider additional motivational strategies to encourage compliance with more burdensome schedules. For example, participants could be more compliant if the research question feels relevant and reminded of the importance of the research they are participating in. Alternatively, participants may prefer reporting on positive health behaviors, depending on whether the study participants were able to or interested in reviewing their data and the relationships found. Due to self-presentation bias, researchers must emphasize that all data are valuable to prevent participants from not responding if they did not engage in positive health-enhancing behaviors such as PA. Importantly, aesthetically pleasing apps that minimize usability issues will likely be critical factors that help maximize compliance in EMA studies examining PA and ST among youth [69].

### Combinations of Different EMA Features and Compliance

In addition to studying the independent impact of EMA design parameters, this study provided unique contributions to the literature by inspecting the combinations of different EMA design features and their associations with compliance among youth. Based on the studies included, we found that daily diaries consistently provided an average of at least 90% compliance regardless of study duration. However, it should be noted that even the more burdensome delivery schemes with multiple EMA prompts per day with 7-14 days of study duration had relatively higher compliance rates, primarily when the prompts were delivered at random times. As the daily EMA prompts increase to 9 prompts or more, the predictable fixed interval yields the highest compliance rate. The caveat is that only 1 EMA study in this category provided the highest compliance rate (99%), but the study consisted of only 1 day of intensive prompting. There was no noticeable interaction between EMA prompting density and study duration, except that longer EMA studies with multiple random prompts per day (<9) had relatively lower compliance (75%).

### Strengths and Limitations of This Review

This review follows a preregistered search strategy, evaluates compliance to EMA by individual and design factors, and assesses an understudied age range. While this review addresses a critical gap in our current knowledge, several limitations exist. First, we did not account for sample size in our analyses. Second, other relevant EMA studies may not be identified in this review based on our preregistered search criteria. This may be particularly important with the high likelihood of some publication biases, with potential studies with low participant compliance remaining unpublished. Third, and similarly, many of the studies excluded or dropped participants’ data with lower compliance from analyses, which may bias our findings to reveal higher study compliance. Nevertheless, EMA studies often have selection bias such that participants who join anticipate being able to be compliant. Our results are still useful as we can still learn from which features are most relevant and predictive of compliance among all these high compliance studies. Fourth, our analyses are based on what was reported in the papers—authors were not contacted to provide additional information when reporting was inadequate. Fifth, the outcome of this study was compliance which we used as a proxy for participant or study burden. Many studies have directly measured burden through usability scales or qualitative studies. Moreover, we did not extract the constructs assessed by the studies through EMA. It is possible that some constructs are more cognitively difficult and cause more burden leading to lower compliance due to participants avoiding completing them. One of our exclusion criteria removed papers that reported on multiple studies; while this simplified data analysis, this may have reduced the information our review gathered on compliance. We excluded papers that reported on multiple studies to maintain consistency in the studies analyzed. Given we were assessing the quality of reporting, authors may have needed to reduce the details of study methods in their papers to meet journal word counts, which obscure insights into compliance trends. Additionally, if the second study described in the paper is an improved iteration of the first study, this contaminates the pooled analyses. Finally, following the CREMAS guidelines, we only extracted data if studies reported on various factors outlined in the checklist, which may exclude extracting potentially relevant details such as what participant training entailed or what attrition rates were.

### Recommendations and Future Directions

This systematic review closely followed each reporting criterion within CREMAS. Additionally, the study explores interactions between some of the more salient factors likely to impact EMA compliance in PA and ST research. Although there was no precise combination of factors that were indicative of higher compliance, especially among delivery schemes with multiple EMAs each day, it should be reiterated that the research design needs to fit the research question and the phenomena of interest—and researchers should think about how to minimize participant burden wherever possible. Importantly, as EMA research continues to increase rapidly in this field, authors must report as much as possible about the design of the study and compensation so future researchers can replicate successful designs.

Beyond what was examined in this review, other factors must be further explored as factors associated with EMA compliance. The first is related to participant training. We did not extract the quality or quantity of EMA training or levels of participant contact in each study—although this was not reported in most studies. Especially with the adoption of mobile devices, researchers must be able to support participants in the case of technical difficulties. Additionally, reporting on difficulties encountered and their impact on compliance would be beneficial, particularly as more studies adopt passive sensing devices to assess PA and ST. Second, more specificity in terms of participant recruitment would also be helpful. How participants were recruited for EMA studies may be particularly important. There may be certain response biases that exist, as participants may not fully understand the high degree of participant burden in community sampling (eg, going into places and spaces to recruit) when compared to participants who were required to reach out to a study team or make an effort to go into a lab or receive in-person training for the study. Moreover, the length of the EMA survey may play a role. We found that the studies with the most EMA prompts per day had high compliance rates, but this may be due to short surveys. The length of time it takes to complete the surveys (number of questions per survey or total completion time) may be a metric for future study. Additionally, greater transparency is needed in reporting, as many papers only report on the number of items that were analyzed when many more items could have been collected. Additionally, this paper was not able to further examine if the amount within the compensation structure matters, as most papers did not disclose the structure. As this may be an important factor, papers should report this information for future analysis. Preregistration on platforms such as the Open Science Framework may help to address this shortcoming. Finally, while the papers all reported on compliance, less than half the studies reported on missing data relationships by assessing if data were missing at random or related to demographic factors. More attention and reporting should be encouraged around patterns of missingness (sporadic, complete attrition after a given point, only during specific windows). Similarly, as compensation is often used as a technique to encourage compliance, the amount of compensation or details on the compensation structure should be reported for future researchers to specifically examine this factor.

### Conclusions

As EMA methodology becomes more prevalent in movement behavior research among youth, it is imperative to understand the potential impact of specific EMA protocol features on study compliance to reduce participant burden and enhance data quality. While this intensive data collection effort involving repeated observations of the participants enables researchers to study the temporal patterns and momentary processes that influence PA and ST, compliance is critical for data quality and validity of the inferences that can be made. Although overall findings did indicate relatively high EMA compliance for PA and ST studies among adolescents and emerging adults, there were some differences based on delivery schemes, study duration, and prompt frequencies. Greater consistency in reporting and more specificity when explaining procedures are required to understand further how EMA compliance could be optimized among this population.

## Appendix

Multimedia Appendix 1 PRISMA checklist.

Multimedia Appendix 2 Extracted data.

## Abbreviations

CREMAS: Checklist for Reporting of EMA Studies
EMA: ecological momentary assessment
PA: physical activity
PRISMA: Preferred Reporting Items for Systematic Reviews and Meta-Analyses
ST: sedentary time
